# Effects of comprehensive intervention on health-related quality of life in patients with chronic hepatitis B in China

**DOI:** 10.1186/1472-6963-13-386

**Published:** 2013-10-04

**Authors:** Jianqian Chao, Long Song, Hua Zhang, Liguo Zhu, Lin Tian, Hui Jin, Pei Liu

**Affiliations:** 1 Department of Medical Insurance, School of Public Health, Southeast University, Nanjing, Jiangsu, China; 2 Jiangsu Provincial Center for Disease Control and Prevention, Nanjing, Jiangsu, China; 3 Department of Epidemiology and Biostatistics, School of Public Health, Southeast University, Nanjing, Jiangsu, China

**Keywords:** Comprehensive intervention, Health-related quality of life, Chronic hepatitis B patients, SF-36, HBV-specific health survey

## Abstract

**Background:**

Hepatitis B virus (HBV) infection is a significant global health problem, especially in China. Chronic liver disease affects health related quality of life (HRQOL). The intervention method to improve HRQOL in patients with hepatitis B has been one-dimensional with inconsistent results. The purpose of this study was to evaluate the effect of comprehensive intervention on health-related quality of life and provide guidance on improving HRQOL for patients with chronic hepatitis B.

**Methods:**

Patients with chronic hepatitis B eligible for our study were randomly selected in three model regions of Jiangsu Province in June 2010. 272 patients were invited and 254 took part, with a refusal rate of 6.62%. Comprehensive intervention included government support, technical guidance from the Chinese Centre for Disease Control and Prevention, standardised medical care, and community involvement. HRQOL before and 1 year after intervention was measured with the Short Form 36 and HBV-specific health surveys. Chi-square test, t-test and multiple linear regressive analyses were used.

**Results:**

After comprehensive intervention, the HRQOL in patients with chronic hepatitis B showed significantly improvements in bodily pain, vitality, social functioning, and mental, as well as physical and mental component score (p < 0.05). Family and social support increased, and financial concerns decreased (p < 0.05). Marital status, duration of illness-related absence from work, education level, family financial status, and health insurance type were important factors affecting HRQOL change between the baseline and final assessment in patients with chronic hepatitis B.

**Conclusion:**

The comprehensive intervention was effective in improving the HRQOL of patients with chronic hepatitis B.

**Trial registration:**

ChiCTR-OCH-12001882

## Background

Hepatitis B virus (HBV) infection is a significant global health problem. An estimated two billion people worldwide have been infected with the hepatitis B virus and more than 240 million have chronic liver infections. About 600000 people die every year due to the acute or chronic consequences of hepatitis B [1]. The prevalence in China is high and includes an estimated 120 million asymptomatic carriers and 6.9 million people infected with HBV, with a prevalence as high as 7.9% [2,3]. Chronic hepatitis B (CHB) increased the risk of liver failure and hepatocellular carcinoma. It imposes considerable economic burden on a family [4]. The incidence rate of hepatitis B ranks first, and the mortality rate ranks third, among all infectious diseases. In 2005, viral hepatitis, AIDS, tuberculosis, and schistosomiasis were listed as the four most important infectious diseases for prevention and control in China [5]. For successful disease control, comprehensive intervention is essential. In 2010, the Ministry of Science and Technology in China launched a major project in the model regions for infectious disease prevention and control [6]. This comprehensive intervention to improve HRQOL for chronic hepatitis B patients was one of the most important study areas for this project.

Recently, increased attention has been given to the patients’ opinions regarding their health status in measures such as health-related quality of life (HRQOL), which is a subjective indicator for evaluation of physiological, psychological and social dimensions. HRQOL has been measured widely in clinical and health service management [7,8]. Certain studies have shown that chronic liver disease affects HRQOL [9-12]. However, few studies have evaluated HRQOL for patients with hepatitis B, and the results from these studies have not been consistent [13-17]. Some studies on HRQOL and related impact factors in patients with hepatitis B patients have been conducted in China [18-22]. These studies have primarily focused on the impact of drug therapy on HRQOL [23,24]. In recent years, some researchers have begun to study the effect of psychological intervention and self-management on HRQOL in patients with chronic liver disease [25,26]. However, there has not been any government-led study in the model regions about the effects of comprehensive intervention on HRQOL and related impact factors in patients with hepatitis B. The prevalence of hepatitis B is high in China and the control measures in some regions are not standardised, with poor effective management and inadequate policy support. Therefore, a multidimensional approach is considered to be important in the treatment and management of these patients. However, few studies have evaluated this in a systematic approach.

The aim of the present study was to evaluate the effects of comprehensive intervention through comparing the changes in patients with chronic hepatitis B patients before and 1 year after comprehensive intervention. We also analyzed the main factors that affect HRQOL. These results would provide guidance on improving HRQOL and comprehensive management of chronic hepatitis B patient.

## Methods

### Study design

This study was conducted in major model regions for infectious disease prevention and control in Jiangsu Province, China from June 2010 to May 2011. The Jiangsu Provincial Centre for Disease Control and Prevention (CDC) initiated and assigned this programme. Patients with chronic hepatitis B eligible for our study were randomly selected in June 2010. The patients were recruited by doctors under the instruction of research assistants. Comprehensive intervention was performed for 1 year. The study had obtained study the approval of the Ethics Committee of the Jiangsu Provincial Centre for Disease Control and Prevention.

### Study participants

Two hundred seventy-two patients were invited and two hundred fifty-four took apart in the end, the refused rate 6.62%. Two hundred fifty-four patients with hepatitis B were involved before intervention, and two hundred twenty-eight patients remained 1 year after intervention began, while twenty-six patients dropped out during intervention with at a rate of 11.4%. The primary reasons for leaving the programme included relocation and patient drop-out during intervention. The study objectives and design were explained to all patients. Afterwards they signed the informed consent.

Criteria for the inclusion of participants were: According to the 2010 Manual of Prevention and Management of Viral Hepatitis in China [27], we included patients with various types’ acute and chronic hepatitis B, liver cirrhosis; liver cancer, and liver transplantation, however, only chronic hepatitis B patients were included in the study. Patients were excluded if they were acute hepatitis B, liver cirrhosis, liver transplantation, liver cancer and other liver diseases not caused by HBV.

Kendall suggested that the sample size of Health-related quality of life was 10 times of the variable number. Our sample size was calculated by it. The variable number is about twenty, so the sample size is about two hundred.

**Figure 1:**
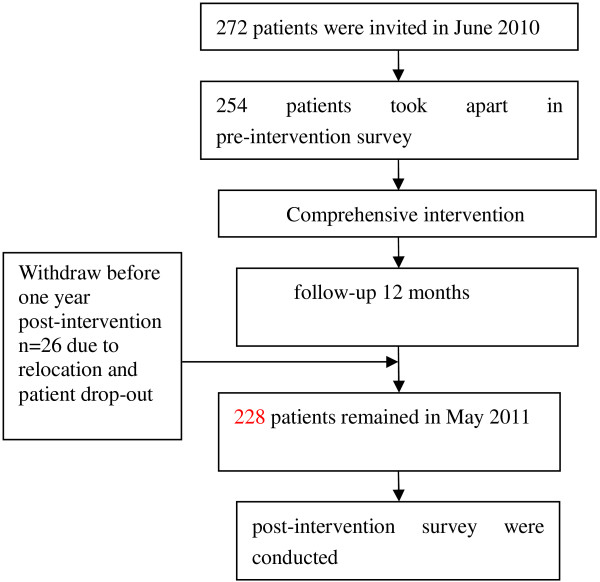
Flow diagram of participants’ progress in the trial.

Two surveys were conducted in June 2010 (pre-intervention) and in May 2011 (post-intervention) by the trained investigators using the questionnaires. Flow diagram of participants’ progress in the trial is showed in Figure 1.

### Comprehensive intervention methods

**Figure 2:**
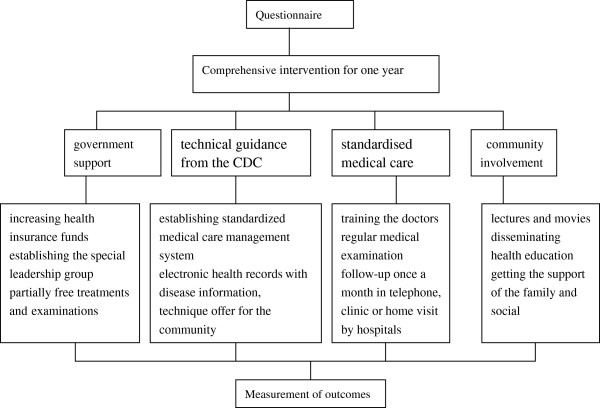
Graphical depiction of the intervention.

Comprehensive intervention was used in our study, and pre- and post-intervention surveys were performed (Figure 2). According to the requirements of the Ministry of Science and Technology for projects on infectious disease prevention in the model regions, the comprehensive intervention from June 2010 to May 2011 included the following: government policy support from a programme funded by the Ministry of Science and Technology in China that supports major and specific projects through increasing health insurance funds and establishing a special leadership group, as well as partially free treatments and examinations; technical guidance from the CDC for hepatitis B patients through establishment of a standardised medical care management system and electronic health records with disease information; the technique offer for the community; implementing standardised medical care through training doctors; regular medical examination(Including the more patients in antiviral medication), with monthly follow-up by telephone or home or clinic visit; and community involvement, such as lectures and films that provide information on hepatitis B, disseminating health education aimed at enhancing patient knowledge and improving HRQOL, as well as gaining family and social support. Therefore, comprehensive intervention involving multiple departments in physiological, psychological and social aspects of care was implemented for chronic hepatitis B patients. The project was performed by the CDC, university and hospitals.

### HRQOL and measurement instruments

HRQOL was measured at months 0 and 12 using two questionnaires: the Medical Outcomes Study 36-item Short-Form Survey (SF-36) Chinese Mainland version 1 [28] and a HBV-specific health survey.

SF-36 consisted of eight dimensions: physical functioning (PF), physical role functioning (RP), bodily pain (BP), general health (GH), vitality (VT), social functioning (SF), emotional role functioning (RE), and mental health (MH). Each scale contained 2–10 items, totalling 36 items, which could be reduced to two summary scores: a physical component score (PCS) and a mental component score (MCS). The PCS included the PF, RP, BP and GH scales, and the MCS included the VT, SF, RE and MH scales. For each question, the raw score was transformed into a scale from 0 (worst possible health state) to 100 (best possible health state). Each scale and total score were calculated in accordance with the corresponding formula [29].

In addition, we designed a HBV-specific health survey to assess HRQOL according to the characteristics of patients with hepatitis B and other related studies. The survey consisted of six scales, including concerns for health and responsibility, family and social support, a psychological tendency toward hostility, concerns over economic status, doctor support, and the sense of being discriminated against. The choices for each item ranged from “all the time” to “none of the time”. For the first four scales, 1, 2, 3, 4, and 5 indicated all of the time, most of the time, some of the time, a little of the time, and none of the time, respectively. For the latter two scales, 1, 2, 3, 4, and 5 indicated none of the time, a little of the time, some of the time, most of the time, and all of the time, respectively. The score from each scale was transformed into a 0–100 score, and higher scores indicated better HRQOL.

The patients completed the questionnaires in outpatient clinics by face-to-face interviewer. Before the investigation, a pilot study was conducted in some patients and the questionnaire was modified according to the results of the pilot study. The reliability and validity of SF-36 are well documented in a range of language versions, including Chinese. Cronbach’s α coefficient of internal consistency for each scale was 0.70–0.90. They all reached the standard of 0.70, which demonstrated the good reliability of the questionnaire. Structural validity of the questionnaire was reflected by factor analysis of principal component analysis and varimax orthogonal rotation, and KMO index was 0.855. Bartlett statistic was χ^2^ = 1142.6, p < 0.001. Factor cumulative contribution of variance was 72.29%. Furthermore, intercommunity of all indexes was >0.5, which indicated good structural validity of the questionnaire. For the surface validity and content validity, relevant experts were invited to discuss the designed questionnaire. They modified the items of inappropriate expression for ensuring its surface and content validity.

### Statistical analysis

The survey data were double-entered by two different people with Epidata 3.1 software (http://www.epidata.dk/). Epidata software was used to check for consistency between the two sets of data entries for quality assurance. Any discrepancies between two people were resolved such that the data entered were a true reflection of that recorded. The quality of data was ensured by setting up logic constraining conditions and valid value ranges of relevant variables. Categorical variables were analysed using χ^2^ test, the measurement data were analysed using *t* test, and a paired t test was used to compare the related scales for HRQOL in patients with chronic hepatitis B at baseline and after the final intervention. Multiple linear regression analyses were used to analyse the factors that affected HRQOL. All analyses were performed in SPSS17.0 (SPSS Inc, Chicago, IL, USA), with 0.05 set as the required level of significance.

## Results

### Patients’ socio-demographic characteristics

The socio-demographic characteristics of the patients were shown in Table 1. Overall, they had a mean age of 43.1 years (range: 13–78), and were mostly male (72.9%). They were mainly middle school or higher (82.8%). 85.4% reported the family economic status fair.

**Table 1:** Socio-demographic characteristics of patients with hepatitis B

**Variable**	**N(228)**	**%**
**Gender**		
Male	167	732
Female	61	26.8
**Age, years** (mean±SD, years)	43.1±13.6	
**Marriage status**		
Married	107	46.9
Other marriage status	121	53.1
**Education level**		
Elementary schools or under	38	16.7
middle school or higher	190	83.3
Family economic status*^1^		
Poor	10	4.4
Fair	197	86.4
Good	21	9.2
**Type of Medical insurance**		
With medical insurance for urban employee/resident	64	28.1
With new rural corporate medical insurance*^2^	155	68.0
Self-pay	9	3.9
**Other chronic disease**		
Yes	191	83.8
No	37	16.2

*^1^Poor: below, Fair: the average income level of the area, Good: above the average income level of the area.

*^2^An insurance type for rural residents.

### SF-36 HRQOl scores

Using the differences in the scores before and after intervention (score after intervention subtracted from the score before) as an index, we compared the changes in HRQOL for each scale in patients with chronic hepatitis B by the paired *t* test (Table 2). After 1 year intervention, the scores for PF, RP, GH, and RE were not significantly improved, but the scores for BP, VT, SF, and MH, as well as PCS and MCS scores were significantly improved (p < 0.05).

**Table 2:** Comparison of SF-36 HRQOL scores in patients with chronic hepatitis B

**Scale**	**Before intervention**	**After intervention**	**t**	***P***
PF	82.71±18.10	81.67±19.84	0.613	0.540
RP	57.03±45.56	58.82±44.28	-0.529	0.598
BP	72.75±16.91	91.93±17.15	-7.388	<0.001
GH	56.25±17.0	58.42±17.60	-1.409	0.161
VT	60.11±19.39	70.74±21.22	-2.805	0.006
SF	75.85±19.43	79.69±19.01	-2.246	0.026
RE	61.66±45.37	64.92±42.71	-0.943	0.347
MH	70.10±17.84	74.73±19.12	-2.902	0.004
PCS	69.26±17.97	72.91±19.86	-2.409	0.017
MCS	66.59±20.78	73.38±21.88	-2.200	0.029

PF: physical functioning; RP: physical role functioning; BP: bodily pain; GH: general health; VT: vitality.

SF: social functioning; RE: emotional role functioning; MH: mental health; PCS: Physical Component score.

MCS: Mental Component score.

### HBV-specific health survey scores

The scores of HBV-specific health survey were showed in Table 3. The scores for family support and concerns over economic status were significantly improved after intervention (p < 0.05). However, there were no significant changes in scores for concerns over health and responsibility, psychological trends toward hostility, doctor support, and the sense of being discriminated against.

**Table 3:** Comparison of HBV-specific HRQOL scores before and 12 months after intervention (mean±SD)

**Scale**	**Before intervention**	**After intervention**	**t**	**p**
Concerns over health and responsibility	86.54±16.93	88.61±15.02	-1.837	0.068
Family and social support	70.38±28.43	78.50±18.35	-3.008	0.003
Psychological tendency toward hostility	98.25±7.37	97.67±5.91	0.899	0.370
Concerns over economic status	66.5±30.69	73.28±28.00	-3.033	0.003
Doctor support	73.34±20.29	74.50±18.59	-0.518	0.605
The sense of being discriminated against	90.31±15.29	89.40±16.25	0.667	0.506

### Factors that affect HRQOL

Multiple linear regression analysis was performed to identify the effect of age, sex, marital status, education level, family economic status, type of medical insurance, chronic disease, years of infection, free treatment status, duration of illness-related absence from work, educational material and activities (first column in Table 4) on the change in the PCS, MCS of SF-36, and HBV-specific health survey for HRQOL (first line in Table 4) between the baseline and final assessment. The following factors affected PCS: marital status, duration of illness-related absence from work, whether educational material was helpful, and educational level. The factors that affected MCS included duration of illness-related absence from work, and marital status. The factors that affected concerns over health and responsibility included duration of illness-related, absence from work and family economic status. The factors that affected the family and social support scale included marital status, whether to participate in publicity educational activities for HBV, and whether the educational materials were helpful. The factors that affected psychological trends in the hostility scale included duration of illness-related absence from work, whether to participate in publicity educational activities for HBV, medical insurance type, and whether the patient had chronic disease. The factor that affected concerns over economic status was duration of illness-related absence from work. The factors that affected the doctor support scale included duration of illness-related absence from work, whether to participate in publicity educational activities for HBV, and whether the educational materials were helpful. The factors that affected the sense of being discriminated against included duration of illness-related, absence from work and whether to participate in publicity educational activities for HBV.

**Table 4:** Multiple linear regression analysis of patients with chronic hepatitis B (standardised partial regression coefficient (β) and 95% confidence interval)

**Impact factors**	**SF 36 PCS**	**SF36 MCS**	**Concerns over health and responsibility**	**Family and social support**	**Psychological tendency toward hostility**	**Concerns over economic status**	**Doctor support**	**Being discriminated against**
**Marriage status**	-0.26(-0.05,-0.48)	-0.34(-0.61,-0.18)		0.39(0.18,0.60)				
**Family economic status**			-0.24(-0.46,-0.01)					
**Duration of illness-related absence from work**	-0.27(-0.49,-0.04)	-0.40(-0.55,-0.12)	-0.30(-0.52,-0.08)		-0.23(-0.45,-0.02)	-0.35(-0.57,-0.13)	-0.29(-0.51,-0.07)	-0.41(-0.59,-0.21)
**Educational material**	0.28(0.07,0.50)			-0.27(-0.50,-0.04)			-0.26(-0.50,-0.01)	
**Educational activities**				0.49(0.26,0.72)	0.33(0.11,0.56)		0.44(0.19,0.68)	0.48(0.28,0.67)
**Type of Medical insurance**					0.27(0.05,0.49)			
**Education level**	0.23(0.01,0.46)							
**Chronic disease**					0.30(0.08,0.51)			

*The impact factors shown in this table had a significant difference (p < 0.05).

## Discussion

There is a shift in focus from specific clinical outcomes to generic ones, such as quality of life which an important means of assessing the results of therapeutic and other interventions. Quality of life is a subjective indicator for evaluations in the physiology, psychology and social dimensions which is widely applied in clinical and health service management [7,11]. The aim of our study was to evaluate the effect of comprehensive intervention on HRQOL in patients with chronic hepatitis B. After the implementation of the comprehensive intervention in the major model regions for infectious disease prevention and control in Jiangsu Province, China, for 1 year, there were improvements in HRQOL. Although the SF-36 scores for PF, RP, GH, and RE did not improved significantly, the scores for BP, VT, SF, MH, PCS and MCS improved significantly after intervention. In the HBV-specific health survey, family and social support was increased, and concerns over economic status were significantly reduced after intervention. However, the concerns over health and responsibility, doctor support, sense of being discriminated against, and psychological trends toward hostility did not change. Therefore, we should further strengthen the support provided by family, society and doctors; supply more care; and reduce social discrimination for patients with chronic hepatitis B.

We compared our results with other studies. Our study showed that at baseline, the scores of patients with chronic hepatitis B for PF, RP, BP, GH, VT, RE, SF, MH were significantly worse that of the general population from the Pan' study [30]. The results from the studies about HRQOL for patients with hepatitis B have not been consistent [13,17,26]. Bondini et al. showed that patients with hepatitis B had similar HRQOL scores as the healthy individuals [13]. When compared with Hong Kong patients, our patients had better scores for BP, GH, and VT scales [31], but they had worse scores for physical health, RP, SF, RE, and psychological functioning scales. The intervention greatly improved both SF and MH in patients with chronic hepatitis B. The scores for the eight HRQOL scales in our patients with chronic hepatitis B were significantly higher than those for patients in Hebei at baseline and after intervention [21]. The patient may develop negative psychological thoughts, as well as physical and social symptoms, because of HBV infection [9]. Certain studies have suggested that proper use of antiviral medications (such as entecavir and lamivudine) could improve health status, prolong lifespan, and improve QOL [9,32]. However, these studies have primarily focused on using drug treatment to reduce clinical complications in patients with hepatitis B. Sharif et al. have studied the impact of psychological intervention on QOL in patients with chronic liver diseases, and have shown that psychological intervention improves scores for the psychological scales [26]. These studies only used a single intervention method (either medication or psychological treatment). The prevalence of hepatitis B is high in China and the control measures in some regions are not standardised, with poor effective management and inadequate policy support. In order to solve these problems, we used comprehensive intervention that included combination of government support, technical guidance from the CDC, standardised medical care, and community involvement, which clearly differs from previous studies regarding the methods applied.

Our study analyzed the factors that affect HRQOL. Marital status affected change in HRQOL between baseline and final assessment. Changes in the scores for PCS and MCS among married patients were larger. However, the changes in family and social support were smaller for married patients, while for widowed/divorced and unmarried patients these changes were larger (β = 0.392, p < 0.05; 0: married, 1: widowed/divorced and unmarried). This may be that the family and social support of widowed/divorced and unmarried patients were less than the married patients and comprehensive intervention could improve the support. For the patients who could not work for illness-related reasons over a longer duration, they had lower changes in the scores for the PCS and MCS; concerns over health and responsibility; and psychological trends toward hostility. When the duration of the illness-related absence from work was ≤3 days, the intervention-mediated positive changes in concerns over economic status, doctor support, and the sense of being discriminated against were larger compared with patients who were absent from work for ≥11 days (β = -0.31, –0.290, –0.405; p < 0.05; 0: ≤3 days, 1: 4–10 days, 2: ≥11 day). A duration of 4–10 days resulted in minimal changes. The patients who believed that educational material was helpful had larger changes in their family, social and doctor support. Patients with higher education levels had greater changes in the scores for the PCS. Higher family economic status resulted in a smaller change for concerns over health and responsibility. Patients with urban employee/resident basic medical insurance had the largest changes in psychological trends toward hostility, which was followed by those with new rural cooperative medical care. Those who pay their own expenses had the smallest changes. Patients without chronic diseases had larger changes in psychological trends toward hostility than those with chronic diseases. To the best of our knowledge, there was little evidence in analyzing the factors influencing the intervention on the change of HRQOL. Based on our analysis, the comprehensive intervention used in this study could reduce psychological trends toward hostility for patients with chronic hepatitis B, as well as improve their family, social and doctor support. In addition, we found that for patients who could not work because of illness, the intervention was more effective for those who were out of work for a shorter duration. Therefore, intervention in chronic hepatitis B patients as soon as possible is recommended and the support for the widowed/divorced and unmarried patients should pay more. Ng et al. showed that information and support they receive at the early stage of the disease determine their adherence to follow up and treatment [33], so it was important to educate doctors on how to communicate the diagnosis and management to patients accurately and sensitively. Some studies have shown that older people have poorer QOL [17,31]. However, Lam et al. and others have shown that younger female patients have lower QOL [31,32,34,35]. Our results did not show an impact of age or sex on the comprehensive intervention for chronic hepatitis B.

Miri et al. showed that the primary vertical route—mothers—has shifted to horizontal routes of HBV transmission [36]. Only the vaccine could not solve the HBV infection problem. Nwokediuko suggested that numerous challenges exist for effective management of chronic HBV infection, particularly in resource-limited regions, such as China, health education was a important method [37]. In order to control the HBV infection, the comprehensive intervention should be considered. Our study evaluated the effects of comprehensive intervention.

Several limitations of the current study should be highlighted. Firstly, the focus of this study was to evaluate the impact of comprehensive intervention on HRQOL in patients with chronic hepatitis B patients in major model regions. We did not evaluate a single intervention method, such as the impact of standardised medical care or community health education. Clearly, this feature of the study represents a possible limitation to future investigations. Secondly, the study did not select other non-model regions as a randomised control group. Thirdly, we did not compare clinical parameters with patients with or without improvement of life quality. The comparations of clinical parameters between before and after intervention were done, there were no significant changes. The reason was that the subjects we studied were chronic hepatitis B, quality of life could change in short time after intervention, but the changes of clinical parameters need a long time. In the future, we will analyze the change and effect of clinical parameters.

## Conclusion

Our study suggest that a comprehensive intervention combining government support, technical guidance from the CDC, standardised medical care and community involvement could be effective for improving the HRQOL of patients with chronic hepatitis B. The results showed that physical pain, VT, SF, MH, PCS and MCS were significantly improved after comprehensive intervention; levels of family and social support were increased; and concerns over economic status were decreased. The impact factors that affected HRQOL for chronic hepatitis B patients included marital status, duration of illness-related absence from work, education level, family economic status, and type of medical insurance. These would provide guidance on improving HRQOL of chronic hepatitis B patient. Further study may better demonstrate potential effect of the comprehensive intervention and impact factors.

## Competing interests

The authors declare that they have no competing interests.

## Authors’ contributions

JQC conducted the data analysis, drafted the manuscript and contributed to subsequent revisions. PL conceived the idea for the study, participated in study design, contributed to the data analysis, the drafting and revising of the manuscript. LS, HZ, LGZ, LT, HJ contributed to implementing the study, analysing the data and editing of the final manuscript. All authors read and approved the final manuscript.

## Pre-publication history

The pre-publication history for this paper can be accessed here:

http://www.biomedcentral.com/1472-6963/13/386/prepub
